# Suicide Surveillance Indicator Framework Quick Stats and Data Tool, cycles 3 and 4

**DOI:** 10.24095/hpcdp.44.2.05

**Published:** 2024-02

**Authors:** Gabriela Williams, Hongbo Liang

**Affiliations:** 1 Public Health Agency of Canada, Ottawa, Ontario, Canada

The Suicide Surveillance Team of the Public Health Agency of Canada (PHAC) is pleased to announce the release of the third and fourth cycles of the Suicide Surveillance Indicator Framework (SSIF) Quick Stats and its Data Tool.

The SSIF update follows government objectives and legislation under the Federal Framework for Suicide Prevention, which requires making statistics publicly available every two years about suicide and related risk factors.

The SSIF Quick Stats includes overall rates and estimates for outcomes and risk and protective factors of suicide. The SSIF Data Tool contains disaggregated data for the four cycles of the SSIF (2017, 2019, 2021 and 2023) by socioeconomic and demographic factors.

These data are derived from administrative sources—such as the Canadian Vital Statistics - Death database (CVS-D), the Discharge Abstract Database (DAD) and the Canadian Hospitals Injury Reporting and Prevention Program (CHIRPP)—and from survey data—such as the Canadian Community Health Survey (CCHS), the General Social Survey (GSS) and the Health Behaviour in School-aged Children (HBSC) study.

Crisis lines are an important indicator of current need of vulnerable populations for access to help. As such, three new sources of data from crisis lines were incorporated into this update of the SSIF: Kids Help Phone, Talk Suicide Canada and the Canadian Surveillance System for Poison Information (CSSPI).

The SSIF Quick Stats and Data Tool can be accessed on the PHAC Infobase website (https://health-infobase.canada.ca/ssif).

